# Preliminary Comparison of Zero-Gravity Chair With Tilt Table in Relation to Heart Rate Variability Measurements

**DOI:** 10.1109/JTEHM.2020.2983147

**Published:** 2020-04-02

**Authors:** Seyedmohsen Dehghanojamahalleh, Vignesh Balasubramanian, Mehmet Kaya

**Affiliations:** Department of Biomedical and Chemical Engineering and SciencesFlorida Institute of Technology 5401 MelbourneFL32901USA

**Keywords:** ECG, HRV, tilt table, zero gravity

## Abstract

Heart rate variability (HRV) measurements are performed using a tilt-table (TT) to diagnose dysfunctionality in the autonomic nervous system (ANS) and the cardiovascular system. To maintain homeostasis, the ANS adapts to body position changes through alterations in sympathetic and parasympathetic responses that can be quantified by extracting time-domain and frequency-domain parameters from the heart rate signal. When body position is changed from supine to erect, a healthy subject’s response also shows changes in ANS activity. However, TT can be unsafe or uncomfortable for elderly or overweight subjects. Furthermore, it may induce anxiety which alters the HRV measurements. This study proposes an alternative strategy to replace the TT with a zero-gravity chair (ZGC). The statistical analysis between HRV parameters from the TT and the ZGC shows that ZGC can be a feasible alternative to TT. Therefore, ZGC can be used as a more convenient, secure, stable and safer option to the traditional HRV analysis with TT.

## Graphical abstract

This study serves as a preliminary proof-of-concept for the replacement of the tilt-table test for heart rate variability with a zero-gravity-chair based test. The zero-gravity chair may be used as a feasible alternative in situations where the use of a tilt table is not safe, convenient or possible.

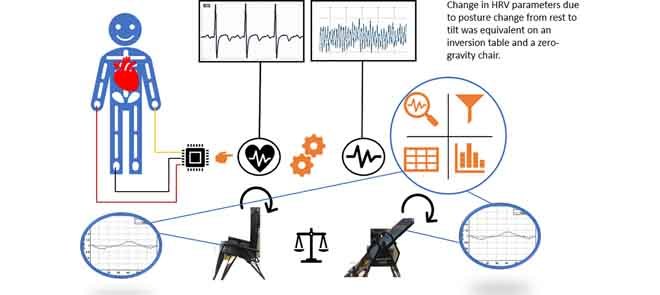

## I. Introduction

Tilt table test has been used to diagnose dysfunctionality in the autonomic nervous system (ANS) or syncope [1]. This test tracks the changes in vital cardiovascular parameters such as blood pressure, heart rate, and heart rate variability during the imposed changes in posture [1]–[5]. TT test, which is also called Head-up Tilt Test (HUT), attempts to create a condition for the subject to induce signs of dysautonomia or syncope [1]–[5]. This condition is caused by changing the body position from supine to erect (vertically standing). Patients with symptoms of dizziness, fainting, and lightheadedness, are the main target group of this test [2].

The procedure is divided into two sections. The first part is to record and monitor the cardiac parameters during the lying position (supine) as the baseline of the test. And the second part begins right after tilting the table angle from 5-15° (horizontal) to 60-80° (vertical) [1], [2]. The signals that are commonly recorded during the procedure include electrocardiograph (ECG), oxygen saturation level (SpO2), photoplethysmography (PPG), and continuous non-invasive blood pressure (CNBP). HRV is one of the most common approaches to evaluating the performance of the ANS [5]–[9] during a TT test. It is an indicator of fluctuations in heart rate. Despite the intrinsic dependence of heart rate on the cardiac pacemaker cells, the ANS can also alter the heart rate through the sympathetic nervous system (SNS) and the parasympathetic nervous system (PSNS) [10]. The PSNS contributes to HRV via the action of acetylcholine through the vagal activity and the SNS controls the release of epinephrine and norepinephrine to alter the heart rate. The SNS is responsible for increases and the PSNS is responsible for decreases in the heart rate [10]–[13]. Therefore, the heart rate is continuously modified due to the interactions between the PSNS and the SNS. It has been previously shown that body position modifies the HRV measurements [14]. Therefore, TT test is frequently used to assess the body’s response to changes in position as an indicator of the ANS activity and its performance [3], [8], [14]. External stimuli such as ambient light, noise, temperature, etc. can alter the HRV results [15], [16].

HRV analysis inspects the variation in the heart rate as a function of time. This can be obtained by 1- recording the cardiac signal, 2- detecting the heartbeats, and 3- extracting the HRV parameters from the beats [3], [8], [17], [18]. There are several ways to detect heartbeats. One of them is to record the electrocardiograph (ECG) signal and identify one of the outstanding waves such as the R wave [19]–[22]. There are also some other signals that can be analyzed to detect the heartbeats including photoplethysmography (PPG) [22]–[25], direct blood pressure signal [26], and ballistocardiography [27], [28]. R peak detection is the most common method in HRV analysis. The time interval between two consecutive R peaks is represented by an R-R interval. Heart rate can be calculated from this parameter.

Sometimes the terminology ‘NN’ is used instead of R-R interval that represents the normal condition of the beats. A set of NNs constructs a heart rate (HR) signal that can reveal some information about the cardiac and the ANS functions. This can be extracted using time-domain analysis and frequency-domain analysis.

The time-domain parameters are extracted from NN intervals by applying mathematical functions such as calculation of standard deviation. The standard deviation of the NN (SDNN) indicates the total HRV power and 5-minute SDNN (SDNN5) represents the total HRV power during a fixed 5-minute recording window. SDNN and SDNN5 are indicators of the ANS cardiac input and overall HRV activity. The root mean square of successive differences (RMSSD) and the standard deviation of the successive differences (SDSD) reflect the power of high-frequency components in the NN data. RMSSD and SDSD are highly dependent on each other and mostly indicate the PSNS activity. NN50 is the number of pairs of successive NNs that differ by more than 50 ms and pNN50 is the proportion of NN50 divided by the total number of NNs. Since pNN50 reflects the beat-to-beat changes in NN intervals, it is considered as an indicator of PSNS activity. A Poincaré plot of the NN intervals can also be plotted. The standard deviation of the distances from each point to the $y =x$ line is considered as SD1 and the standard deviation of the distances from each point to the line $y =x +$ *mean(NN)* is calculated as SD2 [29]. SD1/SD2 ratio represents the autonomic balance and unpredictability of the NN data [29].

Frequency domain parameters are calculated by converting the short-to-midterm NN intervals array into the frequency domain by employing methods such as the Fast Fourier transform (FFT) and dividing the response into frequency bands categorized as ultra-low frequency (ULF: DC to 0.0033 Hz), very low frequency (VLF: 0.0033 and 0.04 Hz), low frequency (LF: 0.04 to 0.15 Hz) and high frequency (HF: 0.15 to 0.4 Hz). Due to the unequal time difference between the NN values, an interpolation method needs to be applied on the NN array prior to performing any frequency-domain parameter extractions. Usually, NN interpolation is performed at 4 Hz.

Table 1 shows a list of the most common time and frequency domain parameters in HRV analysis.

**TABLE 1:** List of Parameters That are Used in the Current Study to Compare the HRV Measurements From a Tilt Table and a Zero-Gravity Chair

Parameter	Domain	Definition
SDNN	Time	Standard deviation (SD) of NN intervals.
SDNN5	Time	5-minute SD of NNs.
RMSSD	Time	The RMS of the successive differences.
SDSD	Time	The SD of the successive differences.
pNN50	Time	The proportion of NN50 divided by total number of NNs.
LF	Frequency	Spectral power in the range of 0.04 to 0.15 Hz.
HF	Frequency	Spectral power in the range of 0.15 to 0.4 Hz.
LF/HF	Frequency	The ratio of power between LF and HF bands.
SD1/SD2	Non-linear	Poincare plot features

**TABLE 2:** Results of the Lilliefors Test Where p < 0.05 Indicates Normality of the Parameter Datasets

Parameter	CON1	CON2	CON3	CON4
LF	0.059	0.5	0.001	0.5
HF	0.5	0.5	0.166	0.5
LF/HF	0.001	0.001	0.001	0.001
SDNN	0.118	0.347	0.191	0.322
SDNN5	0.063	0.45	0.144	0.304
RMSSD	0.2	0.158	0.277	0.261
pNN50	0.5	0.115	0.016	0.357
SDSD	0.203	0.161	0.242	0.261

**TABLE 3:** Rest to Tilt Posture Change Comparison Between ZGC and TT

Feature	ZGC					TT				
	Rest	Tilt	% change in mean	% change in std.	p-value	Rest	Tilt	% change in mean	% change in std.	p-value
LF	0.145 ± 0.034	0.134 ± 0.033	0.08	0.008	0.151	0.150 ± 0.036	0.138 ± 0.028	0.092	0.273	0.098
HF	0.137 ± 0.038	0.149 ± 0.037	−0.08	0.021	0.033	0.125 ± 0.034	0.144 ± 0.040	−0.128	−0.131	0.02
SDNN	55.198 ± 17.952	57.250 ± 16.002	−0.036	0.122	0.472	47.629 ± 14.169	53.659 ± 18.340	−0.112	−0.227	0.028
SDNN5	41.707 ± 14.322	42.800 ± 14.508	−0.026	−0.013	0.681	38.683 ± 11.572	40.979 ± 15.098	−0.056	−0.234	0.302
RMSSD	38.926 ± 14.336	44.071 ± 11.725	−0.117	0.223	0.025	29.132 ± 11.951	39.825 ± 15.013	−0.269	−0.204	<0.001
pNN50	27.500 ± 19.072	55.750 ± 27.025	−0.507	−0.294	<0.001	14.786 ± 14.444	45.393 ± 32.304	−0.674	−0.553	<0.001
SDSD	38.914 ± 14.331	44.058 ± 11.718	−0.117	0.223	0.025	29.119 ± 11.952	39.815 ± 15.009	−0.269	−0.204	<0.001
SD1/SD2	0.388 ± 0.119	0.439 ± 0.122	−0.116	−0.029	0.02	0.331 ± 0.128	0.409 ± 0.113	−0.192	0.139	0.005
LF/HF	1.185 ± 0.576	1.009 ± 0.520	0.175	0.108	0.061	1.333 ± 0.646	1.100 ± 0.588	0.212	0.098	0.132

TT test is traditionally performed by employing a tilt-table (either automated or manual), where the changing angle could also be considered as a physical stimulus to modify the ANS response Additionally, a few studies have shown that anxiety leads to reduced HRV in specific patient groups [30], [31]. Hence, we hypothesized that the uncommon posture induced by the TT test in effects in patients who suffer from anxiety and chronic pain disorders and those that are overweight will result in HRV results that are skewed by anxiety. Any alternative method to address this issue should have a direct impact on the accuracy and reproducibility of HRV in these patient groups for specific diseases.

In order to test our hypothesis, we used a zero-gravity chair to measure the HRV and compared the data with measurements from a conventional TT in this preliminary study. This is the first preliminary study that uses ZGC to perform the TT test as an alternative to the traditional TT. Moreover, we hypothesized that the change in posture induced by the ZGC is more gradual compared to the tilt induced by the TT and this would help mask the skewness introduced by anxiety in the HRV measurements.

The measurement protocol involved the recruitment of participants and testing them on both ZGC and TT. Half of the subjects started with the chair-test first and the other half started with the table-test as the first test. A comprehensive analysis was performed to evaluate the results of these two setups. Finally, it was concluded that the results from the tests performed on the zero-gravity chairs were statistically correlated to the tilt table and therefore, it could be a feasible alternative to the tilt tables.

## II. Materials and Methods

### A. Subjects

This study included 39 volunteers in the range of 18 to 62 years old with an average and standard deviation of 29±7 years. 21 subjects were male, and 18 were female. All the subjects were invited by sending online invitation letters to students, faculty, and staff at Florida Institute of Technology. All the subjects gave signed written informed consent. The testing protocol and the recording lab were approved by the office of compliance and risk management institutional review board (IRB Approval Number: 17-089) at Florida Institute of Technology, Melbourne, FL, USA. Moreover, this study was conducted according to the declaration of Helsinki ethical principles for medical research on human subjects. A questionnaire was given to each subject to collect their family and personal cardiovascular history, medications, diabetic history, alcohol usage, smoking habits, chronic/chest pain, exercise routine and history of neuro/cardiovascular surgery. Subjects were asked to participate in the mornings preferably fasting from midnight or after a very light breakfast. Subjects did not work out before the recording sessions. Subjects were asked to rate their anxiety level on a visual analog scale of 1 to 10 and if the anxiety level was higher than 5, a video of a waterfall with peaceful background music was played for 10 minutes. This continued until the anxiety level went down to values lower than 5. The test was performed in a 24 m^2^ (6 m by 4 m) lab area and the place was kept quiet and at a temperature of 24°C (75°F). The lights were dimmed during the test and were only turned on before and after trials.

### B. Measurement Setup

The regular TT test was performed on an ‘IronMan Gravity 4000’ (World Triathlon Corporation [WTC], Tampa Bay, Florida, USA) tilt table. Two protection straps were placed to limit the tilt range between 10° and 80°. The table supports up to 160 kg (350 lb.) and the straps can handle up to 226 kg (500 lb.). The minimum and maximum supported heights are 145 cm and 198 cm respectively.

The ZGC was a ‘Caravan Global Sports Oversized Zero Gravity Chair’ (Caravan Global, La Mirada, CA, USA). The chair supports up to 150 kg (330 lb.). An adjustable headrest/lumbar support was placed to provide a more comfortable resting position. A cubic pillow (H: 30 cm W: 45 cm L: 35 cm) was placed under the legs during the resting position tests and was removed while performing the tilt position test. Subjects were asked to keep their legs spread out equal to the width of their shoulders and their hands were placed on the armrest during both positions.

Subjects were first placed at the rest position for 5 minutes and the ECG signal was recorded continuously. After that, the table/chair was tilted to the tilt position and the signal was collected for 3 minutes. Then, the test was repeated with the subjects following the same procedure on the table/chair if they had performed the previous test on the chair/table respectively. Subjects spent at least 5 minutes on the platforms prior to the tests and a 2-minute break between the tests. It has been shown that syncope appears 29±19 minutes after tilting [32]. Therefore, it is unlikely for syncope to have happened during this period.

The data, corresponding to the 4 parameters were divided into 4 conditions: 1- Rest position on the chair, 2- Tilt position on the chair, 3- Rest position on the table, 4- Tilt position on the table, which were labeled as CON1, CON2, CON3, and CON4, respectively.

### C. HRV Analysis

The HRV parameters were extracted from the surface ECG lead-I signal. Electrodes were placed on the left shoulder (positive input), right shoulder (negative input) and right side of the neck (as reference). ECG bandwidth was limited to 0.05–150 Hz and the recordings were performed using Medeia HW6D device (Medeia Ltd. Miami, FL, USA 33130) and the HRV parameters were extracted from the ECG signal using the QHRV software (Medeia Ltd. Miami, FL, USA 33130). HRV parameters that have been observed include LF, HF, SDNN, SDNN5, pNN50, RMSSD, SDSD, and SD1/SD2. The interpolation frequency was set at 4 Hz.

Furthermore, the measured LF and HF values were used to calculate the LF/HF ratio. Therefore, the two ratios were calculated as (1) and (2):

\begin{align*} \left ({\frac {LF}{HF} }\right)_{rest}=&\frac {LF_{rest}}{HF_{rest}} \tag{1}\\ \left ({\frac {LF}{HF} }\right)_{tilt}=&\frac {LF_{tilt}}{HF_{tilt}}\tag{2}\end{align*}

The change in body position from rest to tilt causes alterations in all the frequency bands. This is reflected by distinguishable variations in the LF/HF ratio. It should be noted that the normalized units (nu) of the frequency bands must be used to compare the two results. Using equations (3) and (4), normalized LF and HF can be calculated [15].

\begin{align*} {LF}_{nu}=&\frac {LF}{TotalPower-(VLF+ULF)}\approx \frac {LF}{LF+HF}\qquad \tag{3}\\ {HF}_{nu}=&\frac {HF}{TotalPower-(VLF+ULF)}\approx \frac {HF}{LF+HF}\tag{4}\end{align*}

The set of parameters (rest and tilt) in Table 1 helps the physicians to compare the activity of the PSNS and SNS against each other which indicates changes in sympathovagal balance.

### D. Statistical Analysis

The statistical analysis was performed using MATLAB R2017a software (Mathworks, Natick, Massachusetts, USA) on a MacBook Pro that uses 2.6 GHz Intel Core i5 processor (Apple Inc., Cupertino, California USA). The HRV parameters extracted from the chair and the table were analyzed using one-way ANOVA and Pearson Correlation. Initially, changes in HRV parameters due to change in posture from rest to tilt on the ZGC and the TT were analyzed for significant differences using ANOVA. Secondly, the HRV parameters from the TT were compared with the results from the ZGC with data from TT as the reference for both rest and tilt postures. Lilliefors test was used to check the normality of data. Visual inspection of the probability distribution fits of the data was used as the secondary assessment of the normality of data. Lilliefors test was chosen over the Kolgomorov-Smirnov test because the mean and the standard deviation of the population were unknown before performing the test. The significance level was set at 95% for all statistical tests. Eight parameters were considered for analysis from the parameters listed in Table 1 including Normalized LF, Normalized HF, SDNN, SDNN5, pNN50, RMSSD, SDSD, and SD1/SD2. Therefore, the 18 comparison tests were performed as shown in Tables IV and V. These parameters were chosen since they have been used in task force reviews and meta-analysis for short-term HRV parameter extraction [29].

**TABLE 4:** Results of One-Way ANOVA and Pearson Correlation Test Where True Indicates Significant Dependence and False Indicates Independence Between the Observed Parameters From the Rest Position

Test	df	ANOVA p-value	Result	Corr.	p-value	Result
LF	54	0.371	TRUE	0.435	0.021	TRUE
HF	54	0.557	TRUE	0.133	0.501	FALSE
LF/HF	54	0.235	TRUE	0.636	<0.001	TRUE
SDNN	54	0.086	TRUE	0.551	0.002	TRUE
SDNN5	54	0.389	TRUE	0.451	0.016	TRUE
RMSSD	54	0.007	FALSE	0.618	0.001	TRUE
pNN50	54	0.008	FALSE	0.602	0.001	TRUE

**TABLE 5:** Results of One-Way ANOVA and Pearson Correlation Test Where True Indicates Significant Dependence and False Indicates Independence Between the Observed Parameters From the Tilt Position

Test	df	p-value	Result	Corr.	p-value	Result
LF	54	0.571	TRUE	0.64	<0.001	TRUE
HF	54	0.671	TRUE	0.648	<0.001	TRUE
LF/HF	54	0.622	TRUE	0.758	<0.001	TRUE
SDNN	54	0.438	TRUE	0.748	<0.001	TRUE
SDNN5	54	0.647	TRUE	0.711	<0.001	TRUE
RMSSD	54	0.199	TRUE	0.528	0.004	TRUE
pNN50	54	0.243	TRUE	0.711	<0.001	TRUE
SDSD	54	0.244	TRUE	0.711	<0.001	TRUE
SD1/SD2	54	0.122	TRUE	0.646	<0.001	TRUE

## III. Results

### A. Normality Analysis

**FIGURE 1.**
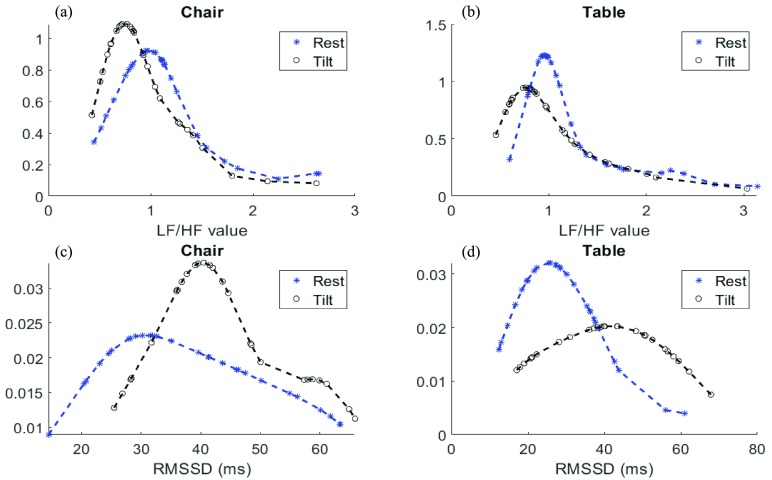
Visual normality inspection of (a) LF/HF ratio on the chair under rest and tilt postures (b) LF/HF ratio on the table under rest and tilt postures (c) RMSSD on the chair under rest and tilt postures (d) RMSSD on the table under rest and tilt postures.

**FIGURE 2.**
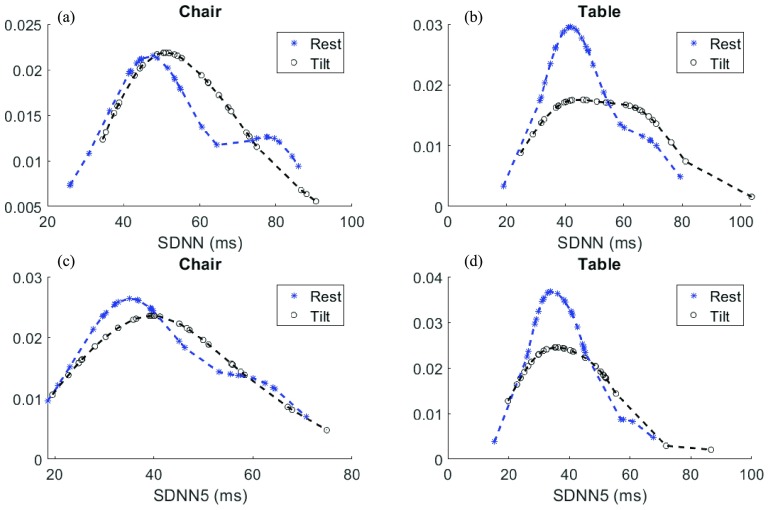
Visual normality inspection of (a) SDNN on the chair under rest and tilt postures (b) SDNN on the table under rest and tilt postures (c) SDNN5 on the chair under rest and tilt postures (d) SDNN5 on the table under rest and tilt postures.

The normality of the parameters was visually inspected by plotting a cumulative distribution fit of the collected data. The distribution of some of the parameters including LF/HF, RMSSD, SDNN, and SDNN5 are illustrated for both chair and table platforms under rest and tilt conditions in Fig 1 and Fig 2. Lilliefors test confirmed the normality assumption for LF/HF ratio (see Table 2) while the Lilliefors test did not fail to reject that the datasets come from a normal distribution for other parameters. However, visual inspection showed that the data were normally distributed, and this was considered significant to identify differences between the estimated parameters from the chair and the table.

### B. ANOVA and Pearson Correlation Analysis

Table 3 lists the comparison of all the features between rest and tilt postures on the chair and the table platforms. It can be seen that there is a significant change in HF, RMSSD, pNN50, SDSD and SD1/SD2 in both the ZGC and the TT trials. Changes in features such as LF, SDNN5 and LF/HF due to the change in posture were insignificant in both the TT test trials. However, change in SDNN due to change in posture from rest to tilt was significant in the TT trial while that was not the case in the ZGC trial. Moreover, the trends in the change in HRV parameters were similar for both the ZGC and the TT trials.

One-way ANOVA (see Tables IV and V) showed that the sample means of the parameters measured in the ‘rest’ condition from the chair were not significantly different for the parameters measured in the ‘rest’ condition from the table. However, one-way ANOVA results for pNN50, RMSSD, SDSD, and SD1/SD2 measured under ‘tilt’ condition from the chair and the table showed that they were significantly different. Moreover, the statistics for the parameters measured under the ‘tilt’ condition showed that the sample means from the chair were not significantly different from the sample means from the table.

Pearson’s correlation analysis showed that all the parameters measured under ‘rest’ and ‘tilt’ conditions had a statistically significant correlation of at least 40% between the parameters that were measured from the chair and those from the table except LF under ‘tilt’ (Tables IV and V).

## IV. Discussion

Variability in the heart rate during a head-up TT test is a common method to evaluate the adaptability of the ANS on body posture. However, there are safety issues and concerns of discomfort that could skew the test results. Therefore, we proposed the use of a ZGC as an alternative to the TT. In order to assess the accuracy of the ZGC for HRV analysis and compare and contrast the similarity of the results from the chair and the table, one-way ANOVA and Pearson correlation were performed on the time- and frequency-domain parameters that were extracted from the recorded signals.

Initial rest-tilt HRV feature changes revealed that the ZGC was able to replicate the changes in all HRV features except that in SDNN as if the TT test were performed on a TT. It has been shown that SDNN is related to parasympathetically active respiratory sinus arrhythmia (RSA) in short term recordings and this effect is modulated by the rate of breathing [29], [33]. From this preliminary analysis, there is evidence to suggest that TT tests on ZGC and TT have subjects breathing differently to one another. The reason for such disparity arises from the difference in tilt positions in the ZGC and the TT. The tilt posture in the TT leads to subjects resting flat on their back compared to resting on an inclined-curved surface on the ZGC and this probably causes them to breathe deeper on the TT than on the ZGC. This discrepancy is not reflected on SDNN5 since the recordings are at most 5 minutes long [29].

Although the SDNN feature was masked by the respiratory changes in the ZGC, changes in features such as RMSSD, pNN50, and HF because of the change in posture are differentiated due to changes in the PSNS activity as in the trial on a TT. Even though RMSSD and HF are affected by RSA [29], the effect of RSA on SDNN is more pronounced due to the deep breathing caused by inherent posture changes on the TT. This result also shows that anxiety disorders issues can be effectively detected using the HF and RMSSD features measured on the ZGC as anxiety disorders cause reduced HF and RMSSD [30]. Furthermore, SDSD is similar to SDNN in the feature domain and it must be noted that the change posture caused a significant change in SDSD measured on the ZGC. The reason for such distinct responses can be attributed to the sensitivity of SDSD to short term variability [29]. Additionally, LF, LF/HF, and SD1/SD2 are dominated by baroreflex activity under specific resting conditions [29], [34]–[36], and the TT test on the ZGC does not seem to cause any anomalies to those measurements.

One-way ANOVA showed that 14 comparisons passed the test and the four parameters that failed were the comparison between pNN50, RMSSD, SDSD and SD1/SD2 in CON2 and CON4. 17 comparisons passed Pearson correlation analysis but normalized HF from CON2 & CON4 failed. One of the reasons that may have caused the hypothesis tests for the comparison of pNN50, RMSSD, SDSD, SD1/SD2 and normalized HF parameters to fail is the length of the recordings. However, FDA-cleared medical devices for HRV measurement usually have similarly limited measurement time. Another critical factor that causes differences between the two measurement set-ups is the activation of PSNS during the rest posture. The ZGC results in a sitting rest posture while the TT results in a standing rest posture. These distinct postures could result in different vagal tones and that is reflected in the preliminary analysis. If the recording time is extended in a future study, then some other parameters such as VLF, ULF, etc. could be also included to understand the long-term effects of the features on the ZGC. Though VLF has been used to simplify the representation of frequency-domain features, VLF measured from 24-hour recordings has been correlated with all-cause mortality, arrhythmia, post-traumatic stress disorder, and inflammation. VLF has been shown to be sensitive to the afferent sensory neurons in the heart and is modulated by PSNS activity among others [29], [33] and a future long-term HRV study on a ZGC will ascertain the effects of the zero-gravity posture on VLF feature.

Another reason for such differences in pNN50, RMSSD, and SDSD can be attributed to gravity. Since the HRV test on ZGC negates gravity under both rest and tilt, the ANS would have adjusted to the effect of zero gravity before the condition changes while the test on TT negates gravity only during the tilt condition which causes the ANS to adjust during the test. In addition to the traditional features, there are other non-linear HRV signal process techniques, such as detrended fluctuation analysis, wavelet transform modulus maxima, Hurst exponents, Higuchi dimension, scaled window variance, entropy estimators and Phase-rectified signal averaging [37]–[39]. The effect of such processing is yet to be ascertained on the ZGC in a future study. However, it should also be noted that the FDA approved devices do not use any of these non-linear feature extraction techniques.

The preliminary study that we conducted shows that the results on a TT can be replicated with short-term recordings on a ZGC for a group of volunteers. However, HRV parameters such as HF, VLF can be reduced in patients with Parkinson’s disease compared to the control group [40]. Similarly, it has been shown that stress associates with elevated LF/HF ratio [41]. Additionally, it has been shown that a single nucleotide polymorphism at position 389 of the $\beta _{1}$ AR gene is associated with TTT [42] and TTT can also be used as a diagnosing procedure for patients with malignant vasovagal syndrome [43] or with unexplained syncope experiences. It should be noted that more specific disease groups have to be recruited in a future study to see if similar results can be replicated on a ZGC for patients that are already suffering from anxiety and chronic pain disorders to ascertain the sensitivity and specificity of the TT test performed on a ZGC.

To sum up, this study serves as a preliminary proof-of-concept for the replacement of the TT test with ZGC based on a few strong statistical correlations between the parameters from the zero-gravity chair and the tilt table. Therefore, the zero-gravity chair may be used as a feasible alternative in situations where the use of a tilt table is not safe, convenient or possible.

## V. Compliance with Ethical Standards

### A. Conflict of Interest

Author Dr. Mehmet Kaya has received research grants from VS Diagnostics (510 Kreag Road Rochester, NY 14534) as part of a consulting project.

Author Seyedmohsen Dehghanojamahalleh declares that he has no conflict of interest.

Author Vignesh Balasubramanian declares that he has no conflict of interest.

### B. Ethical Approval

All procedures performed in studies involving human participants were in accordance with the ethical standards of the institutional and/or national research committee and with the 1964 Helsinki declaration and its later amendments or comparable ethical standards. The testing protocol and the recording lab were approved by the office of compliance and risk management institutional review board (IRB Approval Number: 17-089) at Florida Institute of Technology, Melbourne, FL, USA.

This article does not contain any studies with animals performed by any of the authors.
